# Differences in iNOS and Arginase Expression and Activity in the Macrophages of Rats Are Responsible for the Resistance against *T. gondii* Infection

**DOI:** 10.1371/journal.pone.0035834

**Published:** 2012-04-25

**Authors:** Zhi Li, Zhi-Jun Zhao, Xing-Quan Zhu, Qing-Shi Ren, Fang-Fang Nie, Jiang-Mei Gao, Xiao-Jie Gao, Ting-Bao Yang, Wen-Liang Zhou, Ji-Long Shen, Yong Wang, Fang-Li Lu, Xiao-Guang Chen, Geoff Hide, Francisco J. Ayala, Zhao-Rong Lun

**Affiliations:** 1 State Key Laboratory of Biocontrol, School of Life Sciences and Key Laboratory for Tropical Diseases Control of the Ministry of Education, Zhongshan School of Medicine, Sun Yat-Sen University, Guangzhou, People's Republic of China; 2 State Key Laboratory of Veterinary Etiological Biology, Lanzhou Veterinary Research Institute, Chinese Academy of Agricultural Sciences, Lanzhou, People's Republic of China; 3 Department of Pathogen Biology, Anhui Medical University, Hefei, Anhui, People's Republic of China; 4 Department of Pathogen Biology, Nanjing Medical University, Nanjing, People's Republic of China; 5 School of Public Health and Tropical Medicine, Southern Medical University, Guangzhou, People's Republic of China; 6 Centre for Parasitology and Disease, School of Environment and Life Sciences, University of Salford, Salford, United Kingdom; 7 Department of Ecology and Evolutionary Biology, University of California Irvine, Irvine, California, United States of America; 8 Affiliated Hospital of Ningxia Medical University, Yinchuan, People's Republic of China; Museum National d'Histoire Naturelle, France

## Abstract

*Toxoplasma gondii* infects humans and warm blooded animals causing devastating disease worldwide. It has long been a mystery as to why the peritoneal macrophages of rats are naturally resistant to *T. gondii* infection while those of mice are not. Here, we report that high expression levels and activity of inducible nitric oxide synthase (iNOS) and low levels of arginase-1 (Arg 1) activity in the peritoneal macrophages of rats are responsible for their resistance against *T. gondii* infection, due to high nitric oxide and low polyamines within these cells. The opposite situation was observed in the peritoneal macrophages of mice. This discovery of the opposing functions of iNOS and Arg 1 in rodent peritoneal macrophages may lead to a better understanding of the resistance mechanisms of mammals, particularly humans and livestock, against *T. gondii* and other intracellular pathogens.

## Introduction

Persistent infection is one hallmark of the Apicomplexan protozoan *Toxoplasma gondii*, and it is required for maintaining the parasite's life cycle. This feature and the ability to infect a broad spectrum of warm-blooded vertebrates, including up to 30% of the world's human population, as well as to develop within any nucleated cell type investigated so far, shows *T. gondii* to be one of the most successful obligate intracellular parasites [1]. In most human infected individuals, infection is often asymptomatic and develops into a dormant parasite stage which persists in brain and muscle tissues. *T. gondii* is also a major opportunistic pathogen of fetuses from recently infected mothers, and of immunocompromised patients, i.e. those with organ transplantation and AIDS [2], [3]. In these individuals, the immune system is unable to control the parasite efficiently, leading to unrestricted parasite multiplication and to life-threatening disease.

Rats are naturally resistant to *T. gondii*, in contrast to other rodent mammals such as mice, guinea pigs and hamsters [1], [4], [5]. *T. gondii* does not proliferate in rat peritoneal macrophages *in vitro*, but easily proliferates in peritoneal macrophages of susceptible hosts, such as mice [6]. McCabe and Remington (1986) demonstrated that freshly cultured rat macrophages killed more than 90% of the *T. gondii* ingested and that the surviving *T. gondii* did not replicate when they were observed for up to 72 hrs after ingestion [7]. However, the mechanism of rat macrophage resistance to *T. gondii* remains yet to be determined.

When stimulated with Th1 cytokines [8] or with microbe-derived products [9]–[11], mouse macrophages express the inducible nitric oxide synthase (iNOS), which synthesizes large amounts of nitric oxide (NO) through oxidation of L-arginase. NO is known to be a major effector molecule in macrophage-mediated cytotoxicity and therefore the macrophage-derived NO has been considered a key component of its defense against microbial agents [12], including *Toxoplasma* [13]–[15]. Interestingly, *T. gondii* can easily infect and proliferate in mouse macrophages and reduce their NO production [16], [17].

Arginase shares the same substrate (i.e. L-arginine) with iNOS. Two isoforms of arginase have been identified from macrophages of rat and mouse. Cytoplasmic arginase I and mitochondrial arginase II catalyze the same reaction [18]. Arginase hydrolyzes L-arginine to L-ornithine and urea. L-ornithine favors parasite growth and is the precursor for the synthesis of L-glutamine, L-proline and polyamines via the ornithine decarboxylase (ODC) pathway. Polyamines are essential for the proliferation of cells and parasites [19]–[21]. Furthermore, the potential pathological effects of high NO throughput are limited because arginase competes with iNOS for the same substrate, and it has been established that arginase activity modulates NO production by reducing the availability of L-arginine to iNOS [22], [23].

It has long been known that rat macrophages are naturally resistant to *T. gondii* infection. However, the mechanism of this resistance has not been reported. Many studies have demonstrated that NO can inhibit *T. gondii* proliferation in mouse macrophages after being stimulated with LPS or other cytokines [13], [15]. It has also been shown that in rat and mouse, NOS and arginase activity levels are different in resident peritoneal macrophages [24]. Herein, we raise the questions of whether NO in rat macrophages plays a key role in their resistance to *T. gondii* infection and whether there is any interaction between arginase and iNOS in the rat macrophage that could explain the rat's resistance to *T gondii* infection. The aim of this study is to investigate whether host iNOS and arginase are opposing markers of resistance/susceptibility to *T. gondii* infection in rodent macrophages

## Results

### The levels of iNOS expression and NO production are high in rat peritoneal macrophages compared to undetectable levels in mouse macrophages

**Figure 1:**
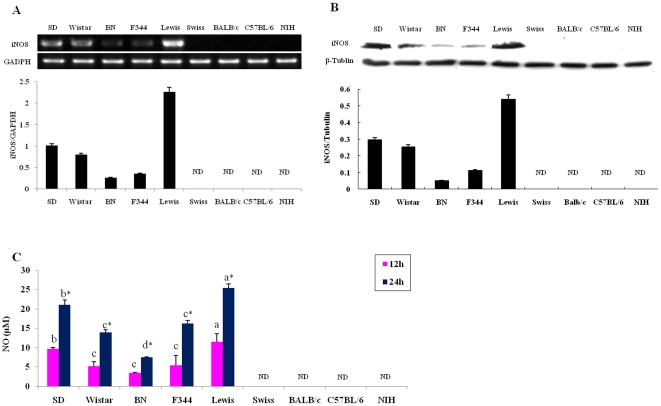
Inducible nitric oxide synthase (iNOS) expression and nitric oxide (NO) production in peritoneal macrophages from 5 rat strains (Sprague-Dawley, Wistar, Brown Norway, F344, and Lewis) and 4 mouse strains (Swiss, BALB/c, C57BL/6, and NIH). (A) (Top) RT-PCR analysis for the expression of iNOS mRNA. (Bottom) Amplicons were densitometrically quantified; bars represent relative amounts of amplified iNOS mRNA normalized against GAPDH. (B) (Top) Western blotting analysis for the expression of iNOS protein. (Bottom) The protein bands were densitometrically quantified and the relative amounts of amplified iNOS protein normalized against tubulin. (C) Comparison of NO production, measured by the Griess reaction, in rat and mouse macrophages. Error bars indicate standard deviations of the measured values (n = 3). One-way ANOVA test to compare any significant differences of NO production among all kinds of groups and different letters above the same bar type indicate significant difference (Duncan, *p*<0.05). Student's *t*-test was used to compare differences between 12 hrs and 24 hrs and statistical differences of NO concentrations between 12 hrs and 24 hrs for each strain were designated by *(*p*<0.05). ND: not detectable (n = 3).

Since there is competition for the substrate (arginine) between iNOS and arginase, we analyzed the level of iNOS expression and NO production in non-activated peritoneal macrophages isolated from 5 strains of rat (Sprague-Dawley (SD), Lewis, Wistar, F344 and Brown Norway (BN)) and 4 strains of mouse (Swiss, BALB/c, C57BL/6 and NIH). Compared to the non-detectable iNOS mRNA expression in mouse peritoneal macrophages, high levels of iNOS mRNA was found in rat peritoneal macrophages (Figure 1A). Among the 5 strains of rat examined, the highest iNOS expression level was observed in the Lewis rat, while the lowest was found in the BN rat. However, iNOS mRNA expression could not be detected in the macrophages from the 4 mouse strains (Fig. 1A). Results from Western blot analysis demonstrated higher expression of iNOS protein in Lewis and SD rats, with lower expression in the other three rat strains, while none was detected in mouse macrophages (Fig. 1B). The concentration of NO in the culture media for the rodent peritoneal macrophages was also measured by the Griess method [25]. In contrast to the undetectable NO in the media from cultivated mouse macrophages, large amounts of NO were detected in the media from cultivation of rat macrophages (25.42±1.08 µM from Lewis rat at 24 hrs) (Fig. 1C). These results are consistent with previous findings that Lewis rat peritoneal macrophages could produce large amounts of NO, while C57BL/6 mouse peritoneal macrophages generated barely detectable traces [26].

### Rat peritoneal macrophages are resistant to the *T. gondii* RH strain while mouse macrophages are susceptible to this parasite

**Figure 2:**
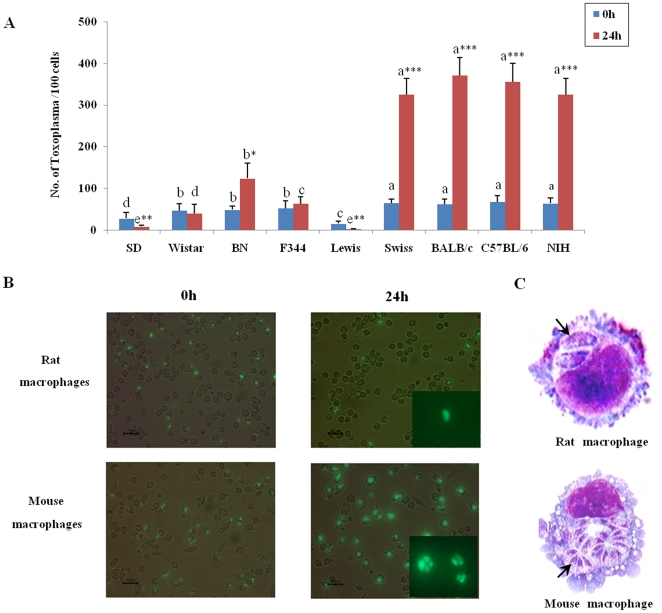
Comparison of *Toxoplasma gondii* proliferation in Sprague-Dawley rat and BALB/c mouse macrophages. (A) Number of *T. gondii* per 100 macrophages counted 0 hr and 24 hrs after infection. Error bars indicate standard deviations of the measured values. One-way ANOVA test to compare any significant differences of parasite numbers among all kinds of groups and different letters above the same bar type indicate significant difference (Duncan, *p*<0.05). Student's *t*-test was used to compare differences between 0 hr and 24 hrs and statistical differences of numbers of infected parasites per 100 cells for each strain between 0 hr and 24 hrs were designated by *(*p*<0.05), ** (*p*<0.01) and *** (*p*<0.001). (B) Analysis of *T. gondii* proliferation in macrophages by fluorescent microscopy. (C) Cells from Fig. 2B stained with Wright-Giemsa 24 hrs after infection (n = 5).

Existing evidence suggests that mouse peritoneal macrophages support the growth of *T. gondii*. We tested this hypothesis by measuring the proliferation of *T. gondii* in non-activated rat and mouse peritoneal macrophages (Fig. 2). Our results indicated that the *T. gondii* RH strain (Type I virulent strain) grew dramatically after 24 hrs infection in mouse peritoneal macrophages *in vitro*; in contrast, a significantly lower number of *T. gondii* were found in rat peritoneal macrophages (Fig. 2A). These results confirm previous studies [6], [7] and demonstrate the comparability of our system. Through fluorescent microscopy and Wright-Giemsa staining of infected cells, we found that after 24 hrs of *T. gondii* infection there were, on average, only one or two parasites in rat macrophages compared to more than 14 parasites in mouse cells, indicating that rat macrophages exhibit high resistance to *T. gondii* (Figs. 2B and 2C). Interestingly, a greater number of parasites were found in the peritoneal macrophages from the BN rat in which we detected a lower level of NO (Fig. 2A). The BN rat has been reported to be more sensitive to other strains of *T. gondii*, such as the Prugniaud strain [27], [28]. Accordingly, we hypothesized that NO could be an important factor involved in rat peritoneal macrophage resistance against *T. gondii* infection. This also supports studies showing the effect of NO against pathogens including *T. gondii* in a mouse model system.

### *T. gondii* proliferation is inhibited in NO-induced macrophages and promoted in NO-decreased cells

**Figure 3:**
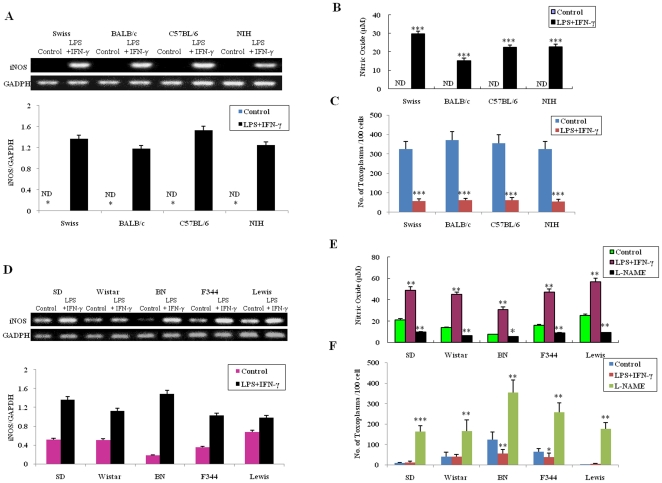
Effect of NO regulation on the growth of *T. gondii* in rat and mouse macrophages. (Top) RT-PCR for iNOS mRNA expression level in mouse (A) and rat (D) peritoneal macrophages treated with LPS+IFN-γ or L-NAME. (Bottom) Amplicons were densitometrically quantified; bars represent relative amounts of amplified iNOS mRNA normalized against GAPDH in mouse (A) and rat (D) macrophages. Comparison of NO production, measured by the Griess reaction, in mouse (B) and rat (E) macrophages treated with LPS+IFN-γ or L-NAME. Number of *T. gondii* per 100 macrophages counted 24 hrs after infection in mouse (C) and rat (F) cells. ND: not detectable. Mean±SEM and significant differences (* *p*<0.05, ** *p*<0.01, *** *p*<0.001) (n = 4).

To characterize the role of NO in resistance to *T. gondii*, we infected rat and mouse cells treated with NO inducer IFN-γ+LPS and iNOS inhibitor N^G^ -nitro-L-arginine methylester (L-NAME). The expression level of the iNOS gene in mouse (Fig. 3A) and rat (Fig. 3D) macrophages activated by LPS+IFN-γ was significantly higher than in non-activated control cells. A significant increase of NO concentration was detected in all groups of mouse (Fig. 3B) and rat (Fig. 3E) peritoneal macrophages treated with LPS+IFN-γ, while a much lower NO concentration was observed in all strains of rat peritoneal macrophages treated with L-NAME (Fig. 3E). The growth of *T. gondii* was significantly inhibited in NO-induced mouse macrophages (Fig. 3C). Although the difference in parasite numbers in the macrophages of SD, Wistar and Lewis rats was not closely associated with LPS+IFN-γ treatment, great discrepancies were found in the peritoneal macrophages of rat strains F344 and BN (Fig. 3F). These results can be attributed to the innate high concentration of NO in Lewis, SD, and Wistar rats which is high enough to inhibit the replication of *T. gondii* and thereby conceal further effects caused by the treatment with LPS+IFN-γ. However, a greater number of parasites were found within the NO-decreased rat peritoneal macrophages treated with L-NAME (Fig. 3F).

### Rats are naturally resistant to *T. gondii*, while mice are highly susceptible

**Figure 4:**
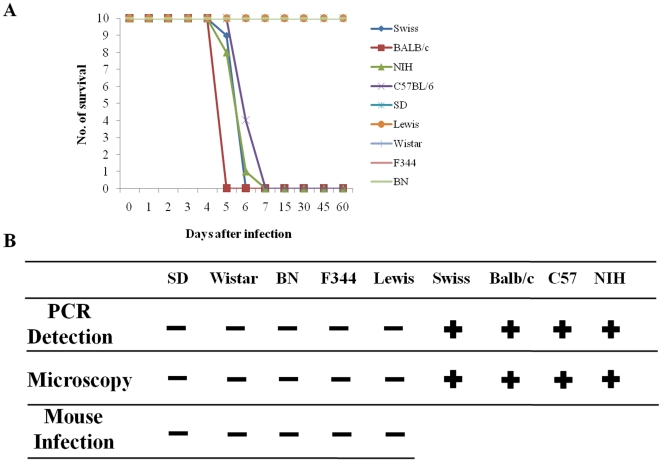
Animal survival curves and detection of parasites after *T. gondii* infection. (A) Survival curve of rat (SD, Lewis, Wistar, F344, and BN) and mouse (Swiss, BALB/c, NIH, and C57BL/6) after 10^6^ (for rat) and 10^5^ (for mouse) RH tachyzoite intraperitoneal infection for 60 days. (B) Detection of *T. gondii* by PCR, microscopy and mouse infection using brain, heart, liver, spleen, lung, and kidney.

We further tested the susceptibility of different strains of mouse and rat to the *T. gondii* RH strain, in order to confirm and extend previous studies [28]–[32]. For two months following inoculation, no deaths were observed in any strains of rat tested, including one-week-old suckling rats infected with 10^6^ tachyzoites of RH strain (intra-peritoneally) (Fig. 4A). Parasites were not detected in the brain, heart, liver, lungs, or kidneys of the infected rats either by inoculation of the organ homogenates into mice or by PCR at 2 months post-infection. However, all strains of mouse tested died from the infection within 3 to 5 days post-inoculation with the same *T. gondii* RH strain. Furthermore, a large number of parasites were found in the above-mentioned organs, taken from the infected mice, by microscopic examination (Fig. 4B). These results further confirm that rats, including newborns, are naturally resistant to the RH strain of *T. gondii*, while mice are highly susceptible to fatal infection [29].

### The level of arginase-1 expression and arginase activity is much higher in mouse peritoneal macrophages than that in rat macrophages

**Figure 5:**
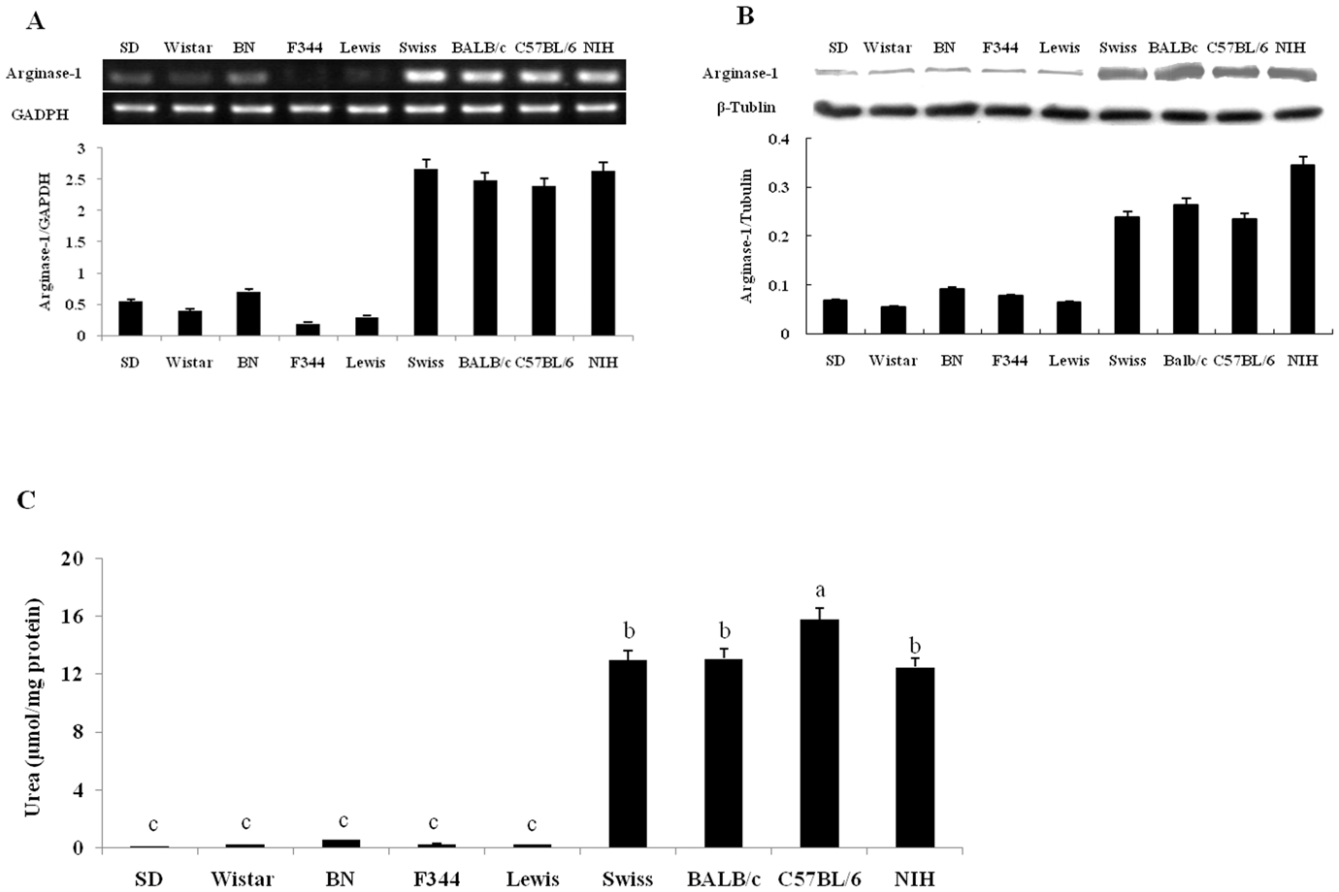
Arginase-1 expression and arginase activity in rat and mouse peritoneal macrophages. (A) (Top) RT-PCR analysis for the expression of arginase-1 mRNA. (Bottom) Amplicons densitometrically quantified; bars represent the relative amounts of amplified arginase-1 mRNA normalized against GAPDH. (B) (Top) Western blotting analysis for expression of arginase-1 protein. (Bottom) Protein bands densitometrically quantified and relative amounts of amplified iNOS protein normalized against tubulin. (C) Arginase activity measured by a colorimetric assay; enzyme activity is the output of urea secreted from lysed macrophages. Error bars indicate standard deviations of the measured values (n = 3). One-way ANOVA test to compare any significant differences of arginase activity among all kinds of groups and different letters above the same bar type indicate significant difference (Duncan, *p*<0.05).

We compared arginase-1 expression and arginase activity in rat and mouse peritoneal macrophages. Our results showed that the level of arginase-1 mRNA expression in macrophages from four strains of mouse was very high, compared to that in macrophages from five strains of rat (Fig. 5A). Western blot results also indicated that the level of arginase-1 protein expression was much higher in mouse macrophages than in rat cells (Fig. 5B). We examined arginase activity in rat and mouse macrophages and found that mouse macrophages produce high arginase activity (15.8±0.9 µmol urea/mg protein from C57BL/6 mouse), compared to rat peritoneal macrophages (0.16±0.037 µmol urea/mg protein from SD rat) (Fig. 5C).

### Levels of iNOS and arginase-1 and the growth of *T. gondii* in peritoneal macrophages from BN, Lewis and BN× Lewis F1 progeny

**Figure 6:**
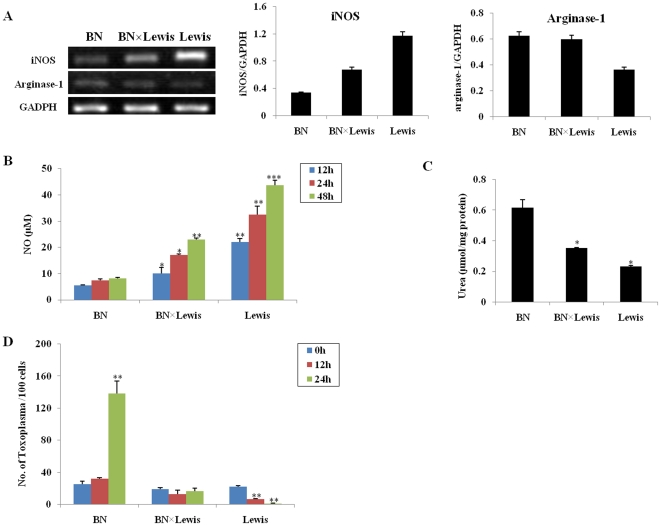
Analysis of iNOS and arginase expression and growth of *T. gondii* in BN, Lewis and BN×Lewis F1 hybrid macrophages. (A) (left) RT-PCR for expression of iNOS and arginase-1 mRNA. Amplicons were densitometrically quantified; bars represent relative amounts of amplified iNOS (middle) and arginase-1 (right) mRNA normalized against GAPDH. (B) NO production measured by the Griess reaction at 12 hrs, 24 hrs, and 48 hrs after infection. (C) Arginase activity measured by a colorimetric assay; enzyme activity is the output of urea secreted from lysed macrophages. (D) Number of *T. gondii* per 100 macrophages at 1 hr, 12 hrs and 24 hrs after infection. Mean ± SEM and significant differences (* *p*<0.05, ** *p*<0.01, *** *p*<0.001) (n = 3).

Our previous data show that among the 5 strains of rat, the expression level of iNOS is highest in Lewis macrophages and lowest in BN macrophages. We therefore decided to ascertain whether any difference in mRNA expression level of iNOS and arginase-1 occurs between BN, Lewis and the F1 progeny of BN× Lewis. The iNOS expression level and NO concentration in the peritoneal macrophages from F1 progeny of BN×Lewis was significantly lower than that of Lewis but higher than that in BN rats. Furthermore, the arginase activity in BN×Lewis was higher than that of Lewis but lower than that in BN rats (Figs. 6A, 6B and 6C). We then examined the growth rate of *T. gondii* RH strain in the peritoneal macrophages from the F1 progeny of BN×Lewis, and found that the number of parasites in the F1 peritoneal macrophages was significantly higher than those from Lewis rats but much lower than those from BN rats. From 0 hr to 48 hrs post-infection, compared to the high levels of growth of *T. gondii* in BN rat macrophages and the absence of *T. gondii* in Lewis rat macrophages, there was no significant difference in the parasite numbers at 0 hr, 12 hrs and 48 hrs after infection (*p*>0.05), indicating that the ability to restrict parasite growth in the F1 progeny of BN×Lewis is higher than in BN but lower than in Lewis (Fig. 6D).

### Proliferation of *T. gondii* is restricted in arginase-inhibited mouse macrophages treated with norNOHA

**Figure 7:**
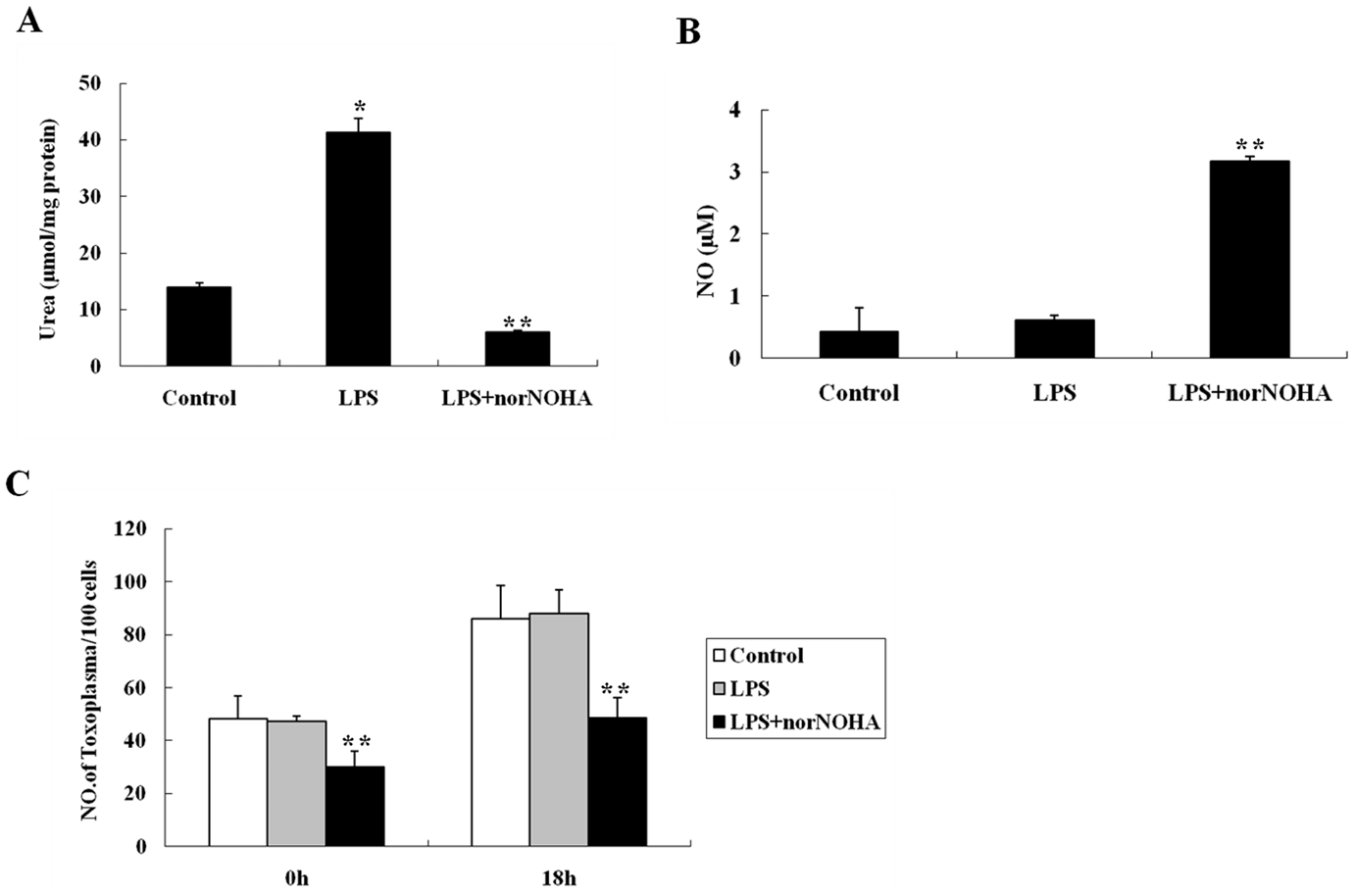
Effect of arginase inhibitor norNOHA on the NO production and *T. gondii* growth in BALB/c mouse peritoneal macrophages. (A) Arginase activity in mouse peritoneal macrophages treated with LPS (0.5 µg/ml) only or LPS (0.5 µg/ml)+norNOHA (500 µM) for 24 hrs, measured by a colorimetric assay; enzyme activity is the output of urea secreted from lysed macrophages. (B) NO production measured by the Griess reaction in mouse macrophages treated with LPS (0.5 µg/ml) only or LPS (0.5 µg/ml)+norNOHA (500 µM) for 24 hrs. (C) Number of *T. gondii* per 100 macrophages counted at 24 hrs after infection in LPS (0.5 µg/ml) only or LPS (0.5 µg/ml)+norNOHA (500 µM) treated mouse macrophages. Mean ± SEM and significant differences (*p<0.05, ** *p*<0.01).

Given that arginase activity in mouse macrophages is very high, we wanted to investigate the growth of *T. gondii* in mouse macrophages in which arginase activity is inhibited by norNOHA. norNOHA was shown by us, both *in vitro* and *in vivo*, that it had no effect on the growth of *Toxoplasma* (Data not shown). Figure 7A shows that arginase activity is significantly decreased in LPS+norNOHA co-treated cells, compared to those LPS-treated only or to control cells. We then found that NO concentration was significantly higher in LPS+norNOHA co-treated mouse macrophages, compared to the low NO level in the LPS-treated only or control cells (*p*<0.01). There is no significant difference in NO level between LPS-treated and control cells (*p*>0.05). These data showed that when arginase activity was inhibited, it could promote NO production (Fig. 7B). We further demonstrated that, in contrast to control cells at 0 hr infection, the number of *T. gondii*/100 cells was significantly decreased in LPS+norNOHA co-treated cells (*P*<0.01). At 18 hrs post-infection, the number of *T. gondii* per 100 cells was also significantly lower in LPS+norNOHA co-treated macrophages, compared to LPS-treated only or control cells. These results showed that the inhibition of arginase activity reduced the infection and proliferation of *T. gondii* in mouse macrophages (Fig. 7C).

## Discussion

Previous research has shown that rat peritoneal macrophages do not support the multiplication of *Toxoplasma gondii in vitro*, but those of mice do [6], [7]. Some explanations have been suggested regarding the mechanism that accounts for this difference, but it is far from understood. A large number of reports have demonstrated that NO is a major effector molecule for macrophage-mediated cytotoxicity in mouse macrophages and is a key anti-pathogen factor used by the infected host to control progression of intracellular pathogens including *Toxoplasma* [13], [33]–[36]. We speculated whether there would be any difference in NO between mouse and rat resident macrophages. Our results show that rat peritoneal macrophages express a high level of iNOS and produce much more NO although difference was found within the strains of rats, whereas NO is undetectable in mouse macrophages, which indicates that NO could be an important factor accounting for the resistance of rat peritoneal macrophages against *T. gondii* infection. We have shown that the number of tachyzoites is significantly higher in rat macrophages treated with L-NAME than in control cells, while the proliferation of *T. gondii* is obviously inhibited in the rat or mouse macrophages treated with LPS+IFN-γ. These data demonstrate that a high concentration of NO in rat peritoneal macrophages is closely associated with their resistance to *T. gondii* infection, supporting our hypothesis that NO in rat macrophages is linked to the resistance to *T. gondii* infection, as implied in published results regarding mouse activated macrophages [14], [16], [33], [37], [38].

Macrophages have been considered one of the key cells for distribution of *T. gondii* to other organs after infection [39], and therefore are suggested to play a part in the natural resistance of rats against the parasite. We have confirmed the fact that rats, even newborns, are naturally resistant to the RH strain of *T. gondii*, while mice are highly susceptible to its fatal infection. Results from the analysis of genetic recombination between BN and Lewis rats, and their F1 progeny, have revealed that a major locus on chromosome 10, called *Toxo1*, mediates resistance to *T. gondii* infection [27]. It was suggested that *Toxo1* is associated with the ability of the macrophage to impede the proliferation of the parasite in the parasitophorous vacuole [27]. We found that the number of tachyzoites of *T. gondii* RH strain in the peritoneal macrophages of the F1 progeny of BN×Lewis was significantly higher than those from Lewis rats but much lower than those from BN rats. Our results also showed that the iNOS expression level and NO concentration in the peritoneal macrophages from the F1 progeny of BN×Lewis was significantly lower than in Lewis rats, but higher than in BN rats. When considering the studies on the *Toxo1* locus [27], we note that the iNOS gene is also located on chromosome 10 (GenBank #24599). From our studies, we suggest that the *Toxo1* locus is likely to be associated with the iNOS gene although additional research will be needed in order to ascertain this matter.

Why is NO so much higher in rat macrophages than in mice? It is well documented that iNOS is responsible for most of the NO production from L-arginine in rodent macrophages [40]. Arginase shares the same substrate (*i.e.* L-arginine) with iNOS and has crucial roles in the host immune system [41], [42]. Arginase 1 (Arg 1) has been induced in alternatively activated macrophages (AAMs) and function in part to suppress NO production in intracellular infection [33]. Arginase 1 hydrolyzes L-arginine to urea and L-ornithine, which are the precursors for the synthesis of polyamines via the ornithine decarboxylase (ODC) pathway. Polyamines promote parasite proliferation due to their inhibition of iNOS expression [43] and because of the inability of *T. gondii* to convert arginine to putrescine, polyamines from the host cell are extremely important course for the growth of this parasite [44]. In fact, because arginase utilizes the same substrate as iNOS, arginase activity can decrease NO production by reducing the availability of L-arginine to iNOS [19], [22], [23], [45].

In order to understand the reason behind the distinctive differences in NO concentration between non-activated peritoneal macrophages of rat and mouse, we analyzed the gene and protein expression of Arg 1 in the peritoneal macrophages from rat and mouse strains. The higher expression level of Arg 1 was accompanied by lower expression of iNOS in the macrophages of mouse strains and, *vice versa*, lower expression of Arg 1 was accompanied by higher expression of iNOS in the rat peritoneal macrophages. Arginase activity in the peritoneal macrophages of BN×Lewis F1 progeny is higher than that in Lewis but lower than that in BN rats. When arginase activity in mouse peritoneal macrophages was reduced by the inhibitor norNOHA (N^ω^-hydroxy-nor-L-arginine), NO production was significantly increased, resulting in the growth inhibition of *T. gondii*. It is likely that substrate competition of these enzymes occurring in the rodent peritoneal macrophages regulates the growth of *T. gondii* by means of NO concentration in the cells. The higher activity of Arg 1 in mouse macrophages will use more arginine to produce more polyamines, which promote the growth of *T. gondii* [44], [46]. In rat macrophages, most of the arginine is used by high iNOS activity to produce more NO [24], which is a harmful molecule for the parasites within the cells. By knocking out the arginase gene from mouse strains, it has been demonstrated that the deletion of Arg 1 significantly prolongs the survival of hosts during *T. gondii* and *Mycobacterium tuberculosis* infections, because more arginine is available to produce NO [33].

In conclusion, our results demonstrate that the different expression levels of iNOS and Arg 1 in rodent peritoneal macrophages work together to determine the resistance and susceptibility to *T. gondii* RH strain infection. High iNOS and low Arg 1 expression level in the rat peritoneal macrophages result in the natural resistance to *T. gondii* infection. In contrast, low iNOS and high Arg 1 expression level in mouse peritoneal macrophages allow the growth of *T. gondii*. The present study highlights the NO-dependent immunity to *T. gondii* and the opposing roles of iNOS and Arg 1 to the growth of the parasite in rat macrophages. These findings provide insights towards understanding the mechanisms used by mammals against infections of *T. gondii* and other intracellular pathogens. Furthermore, the differences in iNOS and Arg 1 activity between individual inbred lines of mice and rats indicates that this mechanism may also account for variation in susceptibility of individuals within natural populations of mammalian species. Further study of these processes may lead to a better understanding of the mechanisms of parasite virulence and host resistance. Such an understanding is likely to improve our ability to develop drugs against *T. gondii*.

## Materials and Methods

### Ethics Statement

All animals were treated in strict accordance to the guidelines for the Laboratory Animal Use and Care from Chinese CDC and the Rules for Medical Laboratory Animals (1998) from Ministry of Health, China, under the protocols approved by National Institute for Communicable Disease Control and Prevention and Laboratory Animal Use and Care Committee of Sun Yat-Sen University under the licenses of 2010CB53000.

### Animals

We used five rat strains, Sprague Dawley (SD), Wistar, Brown Norway (BN), Fischer 344 (F344) and Lewis (6 to 8 weeks old, weight 150∼200 g) and 4 mouse strains, BALB/c, C57BL/6, NIH and Swiss mice (6 to 8 weeks old, weight 25∼30 g). All BN, F344 and Lewis were purchased from Vital River Laboratories (Beijing, China); the other rat and mouse strains were purchased from the Experimental Animal Center of Sun Yat-Sen (Zhongshan) University. All animals were maintained in a pathogen-free room at the School of Life Sciences, Sun Yat-Sen University following the university policy.

### BN× Lewis F1 progeny

BN **(♀)**× Lewis **(♂)** F1 hybrid rats were generated in our laboratory, which were viably fertile, normal in size and did not display any behavioral or physical abnormalities. F1 individuals were identified by a black and white pattern on the underside.

### Parasites

The *Toxoplasma gondii* RH-GFP strain was kindly provided by Dr. X.N. Xuan of the National Research Center for Protozoan Diseases, Obihiro University, Obihiro, Japan, generated as described [47]. For the purification of tachyzoites, *T. gondii* and host cell debris were harvested from the peritoneal cavities of BALB/c mice by injection of ice cold D-Hanks on day 3 after infection. The solution containing *T. gondii* was centrifuged at 40×g for 5 min at 4°C to discard host cells and fragments. The supernatant was centrifuged at 1350×g for 10 min at 4°C, and then suspended in RPMI-1640 medium (GIBCO Laboratories, USA) with 10% fetal bovine serum (FBS) for further use after counted.

### Peritoneal macrophage isolation and cultivation

Animals, sacrificed by carbon dioxide (CO_2_), were injected intraperitoneally with 5 ml (mouse) or 15 ml (rat) ice cold D-Hank's solution containing 100 U of penicillin and 100 µg of streptomycin per ml and then peritoneal cells were harvested and separated by centrifugation at 250×g for 10 min at 4°C. The cells were washed by D-Hank's solution and centrifuged with the same procedure. Finally, cells were suspended in RPMI-1640 medium with 10% FBS and left to adhere for 2 hrs at 37°C in an incubator containing 5% CO_2_ and 95% air. Non-adherent cells were removed and fresh medium was added. Macrophages were cultured overnight and then used for further experiments. Rat peritoneal macrophages were incubated with or without lipopolysaccharide (LPS; 10 µg/ml, Sigma, St. Louis, USA) plus IFN-γ (1 µg/ml, Sigma, USA) or with the NOS specific inhibitor N^ω^ –nitro-L-arginine methyl ester (L-NAME; 10 mM; Sigma, St. Louis, USA); mouse macrophages were incubated alone, or treated with LPS (10 µg/ml) plus IFN-γ (1 µg/ml) or with the arginase inhibitor N^ω^-hydroxy-nor-L-arginine (nor NOHA) (500 µM, Sigma, USA) plus LPS (0.5 µg/ml).

### Parasite infection and detection in animals

Five rat strains (SD, Wistar, BN, F344 and Lewis) and 4 mouse strains (Swiss, BALB/c, C57BL/6 and NIH) were injected intraperitoneally with 10^6^ (for rat) or 10^5^ (for mice, taking into account the body size of the mouse compared to the rat) *T. gondii* RH strain tachyzoites, then observed to construct an animal survival curve. Animal organs (brain, heart, liver, spleen, lung and kidney) were isolated, 4 days (mouse, before death) and 60 days (rat) after infection, for detection of *T. gondii* through microscopy, mouse infection and PCR detection. For the mouse infection, organs (brain, heart, liver, spleen, lung and kidney) from *T. gondii* infected rat 60 days after infection were collected and homogenated respectively, then 0.2 ml homogenate of each organ was injected intraperitoneally to BALB/c mouse. 5 mice were used for each organ homogenate. For the PCR detection, total DNA was extracted from animal organs including brain, heart, liver, spleen, lung and kidney according to the manufacturer's instructions. A 529 bp fragment was amplified from the DNA template using the following primers [48]: 5′-CGCTGCAGGGAGGAAGACGAAAGTTG-3′ and 5′-CGCTGCAGACACAGTGCATCTGGATT-3′. Amplified DNA products were separated on 1% agarose gel and photographed using an electronic documentation system (Biostep, Germany) after staining with ethidium bromide.

### Determination of *T. gondii* intracellular multiplication

Rat or mouse macrophages were challenged with *T. gondii* RH strain tachyzoites at ratio 1∶1 (*T. gondii*/macrophage). Extracellular *T. gondii* were then washed out after 1 hr incubation together, at which the time point was defined as 0 hr for the start of the experiment. Thereafter, the cells were observed with an inverted fluorescence microscope or stained with Giemsa at the desired time. The numbers of *T. gondii* were counted in 100 host macrophages and an average determined.

### Measurement of iNOS and arginase activity

Nitrite content as a reflection of NO production was determined by the Griess reaction as described [25]. Briefly, 100 µl supernatant or standard solution (NaNO_2_) were incubated in triplicate with 100 µl of Griess reagent (0.5%sulfanilamide, 0.05% naphthyldiamine dihydrochloride in 5% H_3_PO_4_) for 10 min. The plates were read at 550 nm in an ELISA reader (Multiskan MK3, Thermo Labsystems, Finland).

Arginase activity of purified macrophage was measured by a colorimetric method as described [49]. Briefly, 10 mM MnCl_2_ and 0.5 M L-arginine were successively added to macrophage lysates for 1 hr at 37°C. The reaction was stopped by addition of an acid solution (H_2_SO_4_∶H_3_PO_4_∶H_2_O = 1∶3∶7), and the urea generated by arginase was analyzed by addition of α-isonitrosopropiophenone at 100°C for 45 min. The colored product was quantified by absorption at 550 nm in an ELISA reader. Arginase activity was determined as the amount of urea produced from total protein of peritoneal macrophages.

### mRNA analysis

Total RNA from treated and non-treated macrophages was extracted using Trizol Reagent (Invitrogen, Carlsbad, USA) according to the manufacturer's instructions. Total RNA was converted to cDNA using a set of oligo (dT) primer and SuperScript™ III First-Strand Synthesis System (Invitrogen, Carlsbad, USA). cDNA (1 µg) was used as a template for amplifying iNOS, arginase-1 and GAPDH (as internal standard) genes by PCR using the following primers: arginase-1, 5′-AAG AAA AGG CCG ATT CAC CT-3′ and 5′-CAC CTC CTC TGC TGT CTT CC-3′, 201 bp; rat-iNOS, 5′-CTA CCT ACC TGG GGA ACA CCT GGG-3′ and 5′-GGA GGA GCT GAT GGA GTA GTA GCG G-3′, 442 bp; mouse-iNOS, 5′-GCC TCG CTC TGG AAA GA-3′ and 5′-TCC ATG CAG ACA ACC TT-3′, 499 bp; GADPH, 5′-AAT GCK TCC TGY ACC ACC AAC TGC-3′ and 5′-TTA GCC AWA TTC RTT GTC RTA CCA GG-3′, 513 bp [10]. For semiquantitative PCR, the cycle was: 94°C for 1 min, 60°C for 1.5 min, and 72°C for 1.8 min. For arginase-1, rat-iNOS, mouse-iNOS, 27 cycles were used, but for GAPDH, only 20 cycles. Amplified DNA products were separated on 1% agarose gel and photographed using an electronic documentation system (Biostep, Germany) after staining with ethidium bromide. Signal intensity was quantified using Gelix One software.

### Western Blotting

Cells were lysed in SDS loading buffer, fractionated in SDS-PAGE and transferred onto immunoblot polyvinylidene difluoride membrane (Pall, USA). The membrane was probed using rabbit polyclonal iNOS antibody (Thermo, USA) and rabbit polyclonal arginase-1 antibody (Santa Cruz, USA). β-tubulin was stained with antibody (NOVUS, USA) as control. Horseradish peroxidase-labeled secondary antibodies (Cell Signaling, USA) and DAB (3,3′,5,5′-tetramethylbenzidine) Detection Kit (Tiangen, China) were used for antibody detection. Signal intensity was quantified using Gelix One software.

### Statistical analysis

Results are expressed as mean ± SEM. Multiple data comparisons were derived by one-way ANOVA using SPSS 13.0 software (SPSS Inc., Chicago, USA). We used a one-way ANOVA test (Duncan test, *p*<0.05) to compare any significant differences among all kinds of groups for the same time point. Student's *t*-test was used to compare differences between two unpaired samples and statistical differences were designated by *(*p*<0.05), ** (*p*<0.01) and *** (*p*<0.001).
